# FB5P-seq-mAbs: monoclonal antibody production from FB5P-seq libraries for integrative single-cell analysis of B cells

**DOI:** 10.3389/fimmu.2024.1505971

**Published:** 2024-12-17

**Authors:** Sakina Ado, Chuang Dong, Noudjoud Attaf, Myriam Moussa, Agathe Carrier, Pierre Milpied, Jean-Marc Navarro

**Affiliations:** ^1^ Aix Marseille Université, CNRS, INSERM, Centre d’Immunologie de Marseille-Luminy, Marseille, France; ^2^ Paris-Saclay University, Inserm, Gustave Roussy, Tumour Immunology and Anti-Cancer Immunotherapy, Villejuif, France

**Keywords:** B cells, single-cell RNA-seq, antibody cloning, BCR sequencing, antigen specificity

## Abstract

Parallel analysis of phenotype, transcriptome and antigen receptor sequence in single B cells is a useful method for tracking B cell activation and maturation during immune responses. However, in most cases, the specificity and affinity of the B cell antigen receptor cannot be inferred from its sequence. Antibody cloning and expression from single B cells is then required for functional assays. Here we propose a method that integrates FACS-based 5’-end single-cell RNA sequencing (FB5P-seq) and monoclonal antibody cloning for integrative analysis of single B cells. Starting from a cell suspension, single B cells are FACS-sorted into 96-well plates for reverse transcription, cDNA barcoding and amplification. A fraction of the single-cell cDNA is used for preparing 5’-end RNA-seq libraries that are sequenced for retrieving transcriptome-wide gene expression and paired BCR sequences. The archived cDNA of selected cells of interest is used as input for cloning heavy and light chain variable regions into antibody expression plasmid vectors. The corresponding monoclonal antibodies are produced by transient transfection of a eukaryotic producing cell line and purified for functional assays. We provide detailed step-by-step instructions and describe results obtained on ovalbumin-specific murine germinal center B cells after immunization. Our method is robust, flexible, cost-effective, and applicable to different B cell types and species. We anticipate it will be useful for mapping antigen specificity and affinity of rare B cell subsets characterized by defined gene expression and/or antigen receptor sequence.

## Introduction

In the immune response to pathogens and vaccines, antigen-responsive B cells undergo series of cellular and molecular maturation events that are required for long term immune protection and memory (1). On the cellular side, activated B cells divide, migrate, and evolve through distinct intermediate differentiation stages that ultimately give rise to long-lived antibody-producing plasma cells and memory B cells (2). On the molecular side, within each responding B cell, the genetic *loci* encoding B cell receptor (BCR) immunoglobulin heavy (IGH) and light (IGK/L) chains may undergo class switch recombination (CSR) (3) and somatic hypermutation (SHM) (4), providing opportunities for improving the function and affinity of antigen-specific antibodies produced throughout the current and future immune responses (5). In those parallel cellular and molecular evolution processes, every antigen-responsive B cell may generate a diverse progeny of clonally related daughter B cells expressing unique combinations of functional properties and BCR affinities. Thus, methods that enable integrative analysis of B cell phenotype, transcriptome, BCR sequence, and BCR affinity at the single-cell level, are useful tools when studying B cell immune responses (6).

In the past few years, two types of single-cell RNA sequencing (scRNA-seq) techniques have been applied to B cells for integrative analysis of transcriptome and BCR sequence: plate-based scRNA-seq of B cells sorted by flow cytometry, either with full-length (Smart-seq2) (7, 8) or 5’-end sequencing (FB5P-seq) (9); and droplet-based 10x Genomics 5’-end scRNA-seq (10). Both techniques may link B cell transcriptome and BCR sequence with antigen-specificity, provided B cells are incubated with labeled antigen [fluorescent antigen for FACS-based methods (11), DNA-barcoded antigen for droplet-based method (12)] before analysis. However, those approaches are not suitable for B cells expressing low amounts of surface BCR (e.g. plasma cells), and preclude extensive analyses of BCR specificity, affinity and function.

Antibody cloning and production from single FACS-sorted memory B cells or plasmablasts [e.g. protocols described in (13–15)], has been the method of choice for discovering and characterizing naturally occurring antigen-specific antibodies from infected individuals, notably in the fields of HIV (16) and SARS-CoV2 (17) research. Such methods, applied to animal models of protein vaccination or infection, have also contributed to our basic understanding of the role of BCR affinity during germinal center (GC) B cell responses (18, 19). However, in those studies, the cellular characteristics of B cells from which monoclonal antibodies (mAbs) are cloned can only be inferred from the expression of a few surface markers or of fluorescent reporters.

**Figure 1:**
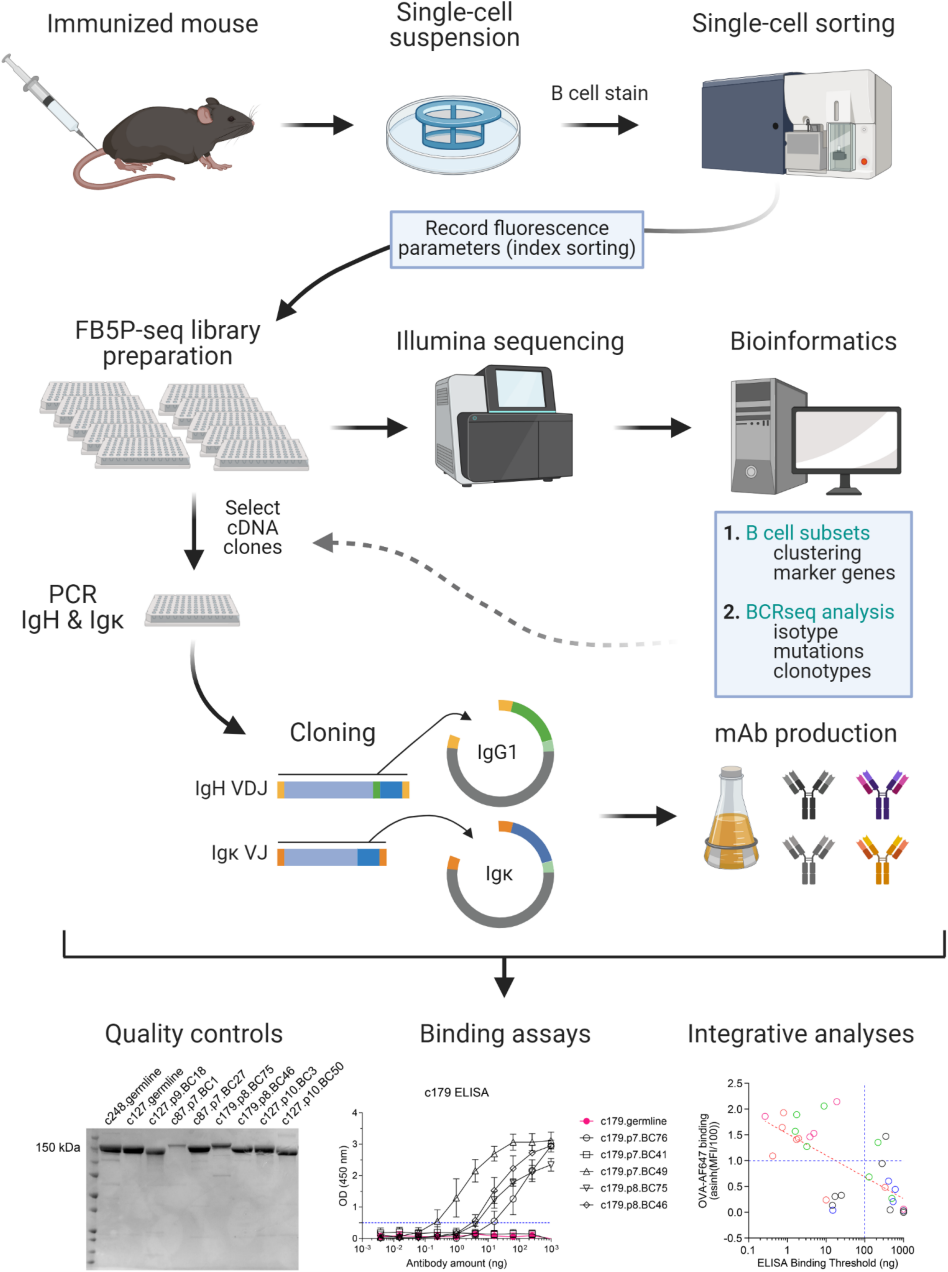
Workflow of FB5P-seq-mAbs. Starting from an immunized mouse, single B cells of interest are sorted by FACS for 5’-end single-cell RNA-seq library preparation with the FB5P-seq protocol (9), recording fluorescence intensity parameters of each sorted cell by index sorting. FB5P-seq libraries from multiple 96-well plates are pooled, sequenced, and analyzed to identify B cell subsets, recover paired BCR heavy and light chain sequences, and integrate both information types. For selected cells of interest, the remaining single-cell amplified cDNA in archived 96-well PCR plates is used as starting material for PCR amplification of IgH VDJ and Igκ VJ sequences, and cloning into mouse IgG1 and Igκ expression vectors, respectively. IgG1 and Igκ expression plasmids are co-transfected into a eukaryotic cell line for production of recombinant mAb in the culture supernatant. After purification, recombinant IgG1/κ mAbs are available for functional assays and integrative analyses. Created with BioRender.com.

Here we describe FB5P-seq-mAbs, a method that bridges plate-based 5’-end scRNA-seq (FB5P-seq) with recombinant monoclonal antibody cloning and production (**Figure 1**), and illustrate its use for characterizing antigen-responding GC B cells after chicken ovalbumin (OVA) immunization in mice.

## Materials and equipment

### Antibodies

**Table:** 

Reagent or resource	Source	Identifier
CD138-BV711	Biolegend	cat.no.142519
CD19-PE-Dazzle594	Biolegend	cat.no. 115553
CD38-PE	Biolegend	cat.no. 356608
CD3-APC-Cy7	Biolegend	cat.no.100222
CXCR4-PerCP-eF710	ThermoFisher Scientific	cat.no. 46-9991-82
GL7-BV421	Biolegend	cat.no.144614
Gr1-APC-Cy7	Biolegend	cat.no.108424
Mouse Fc block	Biolegend	cat.no.101320
OVA-AF 647	ThermoFisher Scientific	cat.no. O34784
HRP-coupled anti-mouse IgG secondary antibody	ThermoFisher Scientific	cat.no. A24512
Anti-ovalbumin (clone 6C8)	Abcam	cat.no. ab17293

### Cell lines, mouse strains, bacteria

**Table:** 

Reagent or resource	Source	Identifier
Aicda-Cre-ERT2 x Rosa26-lox-STOP-lox-eYFP	Le Gallou et al. (20)	
E.Coli JM109	Promega	cat.no. L2005
Expi293TM	ThermoFisher Scientific	cat.no. A14527

### Chemicals, peptides and recombinant proteins

**Table:** 

Reagent or resource	Source	Identifier
1Kb plus DNA ladder	ThermoFisher Scientific	cat.no. 10787018
2N sulfuric acid	ThermoFisher Scientific	cat.no. N600
2-Propanol	Sigma-Aldrich	cat.no. 109634
Acetic acid	VWR	cat.no. 20104.334
Agarose	Life Technologies	cat.no. 16500500
AgeI-HF	New England Biolabs	cat.no. R3552S
Alkaline Phosphatase	New England Biolabs	cat.no. M0525S
Alum	ThermoFisher Scientific	cat.no. 77161
Ampicillin	Sigma-Aldrich	cat.no. A9518
Arachidic acid oil	Sigma-Aldrich	cat.no. A3631
Betaine	Sigma-Aldrich	cat.no. B0300
Boric Acid	Carlo Erba	cat.no. 600641
Bromophenol Blue	Sigma-Aldrich	Cat no. B0126-25G
Bovine Serum Albumin	Sigma-Aldrich	cat.no. 10735078001
BsiwI	New England Biolabs	cat.no. R0553L
Clean NGS beads	Proteigene	cat.no. CNGS-0050
CutSmart Buffer	New England Biolabs	cat.no. B7203S
Dimethyl sulfoxide	Merck	cat.no. 276855-100ML
dNTP	ThermoFisher Scientific	cat.no. 10297018
EDTA	ThermoFisher Scientific	cat.no. 15575020
Ethanol	Sigma-Aldrich	cat.no. M3148-100ML
Ethidium bromide	Merck	cas.no. E1510
Expi293™ medium	ThermoFisher Scientific	cat.no. A1435101
Fetal Calf Serum	Gibco	cat.no. 10270-106
Glucose	Merck	cat.no. 49159-1KG
Glycine	ThermoFisher Scientific	cat no. 15527-013
GoTaq G2 Flexi enzyme	Promega	cat no. M7801
HCl	Dutscher	cat no. 524526
KAPA HiFi ReadyMix	Roche Diagnostics	cat.no. 7958935001
LB Agar	ThermoFisher Scientific	cat.no. 22700025
LB medium	ThermoFisher Scientific	cat.no. 12780052
Live/Dead	ThermoFisher Scientific	cat.no. L34957
Protein ladder	Biorad	cat.no. 1610373
Methanol	Dutscher	cat.no. 412383
MgCl_2_	Sigma-Aldrich	cat.no. M1028
NaCl	Sigma-Aldrich	cat.no. S6546-1L
NEBuffer 2	New England Biolabs	cat.no. B7202
NEBuffer 3.1	New England Biolabs	cat.no. B7203S
Nupage novex 4-12% BT midi gel	ThermoFisher Scientific	cat.no. WG1401BOX
NuPAGE™ MOPS SDS Running Buffer (20X)	Life Technologies	cat.no. NP0001
OVA antigen	InvivoGen	cat.no. vac-stova
OVA stock	ThermoFisher Scientific	cat.no. 77120
PBS 10 X	Life Technologies	cat.no. 14200067
PCR-grade H_2_O	Qiagen	cat.no. 129114
Penicillin-streptomycin	ThermoFisher Scientific	cat.no. 15140122
Polyethylene glycol 3350	Merck	cat.no. P4338-500G
Polyethyleneimine	Sigma-Aldrich	cat.no. 408727-100ML
Protein G beads	Sigma-Aldrich	cat.no. GE17-0618-01
Rnase OUT	ThermoFisher Scientific	cat.no. 10777019
Laemmli SDS-Sample Buffer 4X, Non-Reducing	Cliniscience	cat.no. 10570018-1
Sample Buffer Laemmli 2x Concentrate	Sigma-Aldrich	cat.no. S3401-10VL
SOC medium	Invitrogen	cat.no. 15544-034
Sodium azide	Merck	cat.no. S2002-5G
SuperScript II	ThermoFisher Scientific	cat.no. 18064014
T4 DNA polymerase	New England Biolabs	cat.no. M0203S
Tamoxifen	Sigma-Aldrich	cat.no. T5648-1G
TMB Solution	ThermoFisher Scientific	cat.no. 34021
Tris base	Life Technologies	cat.no. 15504020
Triton X-100	Merck	cat.no. 93443-100ML
Trypan blue	Merck	cat.no. T8154-20ML
Tryptone	Merck	cas.no. T7293-250G
Tween 20	Merck	cat.no. P9416-100ML
Valproic acid	Merck	cat.no. V0033000
XhoI	New England Biolabs	cat.no. R0146L
Yeast extract	Merck	cat.no. Y1625-250G

### Oligonucleotides

**Table:** 

Reagent or resource	Source	Identifier
(dT)30_Smarter	IDT	*Attaf et al.* (9)
ERCC spike-in Mix	ThermoFisher Scientific	cat.no. 4456740
i5 primer S5xx	Illumina	cat.no. FC-131-2001
i7 primers mix	IDT	*Attaf et al.* (9)
i7_BCx primer	IDT	*Attaf et al.* (9)
Multiplex Forward Primers	IDT	**Supplementary Table 1**
Multiplex Reverse Primers	IDT	**Supplementary Table 1**
PCR_Satija	IDT	*Attaf et al.* (9)
Screening Forward Primers	IDT	**Supplementary Table 2**
Screening Reverse Primers	IDT	**Supplementary Table 2**
SmarterR	IDT	*Attaf et al.* (9)
TSO_BCx_UMI5_TATA	IDT	*Attaf et al.* (9)

### Commercial assays

**Table:** 

Reagent or resource	Source	Identifier
High Sensitivity DNA chip analysis	Agilent	cat.no. 5067-4626
High Speed Midi Kit	Qiagen	cat.no. 12643
Nextera XT DNA sample Preparation kit	Illumina	cat.no. FC-131-1096
QIAquick 96 PCR Purification Kit	Qiagen	cat.no. 28181
QIAquick Gel Extraction Kit	Qiagen	cat.no. 28704
Qubit HS DNA test	ThermoFisher Scientific	cat.no. Q32854

### Equipment

**Table:** 

Reagent or resource	Source	Identifier
8-channel micropipette 10 µl	Starlab	cat.no. S7108-0510
8-channel micropipette 100 µl	Starlab	cat.no. S7108-1100
8-channel micropipette 300 µl	Starlab	cat.no. S7108-3300
BD Influx™ Cell Sorter	BD Biosciences	NA
Benchtop plate centrifuge	Starlab	cat.no. N2631-0008
Incubator	Fisher Scientific	cat.no. 12815883
DynaMag™-96	ThermoFisher Scientific	cat.no. 12331D
Nanodrop	ThermoFisher Scientific	cat.no. ND-ONE
p10 micropipette	Starlab	cat.no. S7100-0510
p1000 micropipette	Starlab	cat.no. S7110-1000
p200 micropipette	Starlab	cat.no. S7100-2200
Plate reader	BMG LABTECH	NA
Shaker	VWR	cat.no. 444-4227
Sonicator	Kinematica	cat.no. HS1200 E
Thermal Cycler	ThermoFisher Scientific	cat.no. A24811
UV light	Dutscher	cat.no. 5207326
Vortex	Dutscher	cat.no. 79008

### Consumables

**Table:** 

Reagent or resource	Source	Identifier
0.22-micron steritop filters	Millipore	cat.no. SCGPT01RE
0.3 ml syringe	Dutscher	cat.no. 324826
1.5 ml tube	Eppendorf	cat.no. 30108051
10 ml serological pipette	Becton dickinson	cat.no. 357551
10 μl filter tips	Starlab	cat.no. S1120-3810
1000 μl filter tips	Dutscher	cat.no. 134000CL
15 ml tube	Dutscher	cat.no. 352096
200 μl filter tips	Sarstedt	cat.no. 3070279
25 ml serological pipette	Sarstedt	cat.no. 86.1685.001
30 KD amicon	Merck	cat.no. UFC503024
5 ml FACS tubes	Dutscher	cat.no. 352054
5 ml serological pipette	Sarstedt	cat.no. 86.1253.001
50 ml tube	Sarstedt	cat.no. 62.547.254
70 µm cell strainer	Sarstedt	cat.no. 83.3945.070
8-tube PCR strip	Merck	cat.no. EP0030124359
96 round bottom	Dutscher	cat.no. 353077
96-well ELISA plates	ThermoFisher Scientific	cat.no. 467320
96-well PCR plates	ThermoFisher Scientific	cat.no. 4483485
Adhesive film	Life Technologies	cat.no. 4306311
Aluminum foil	VWR	cat.no. 291-0045
Chromatography columns	Sigma	cat.no. 7311550
Dissection scissors	Dutscher	cat.no. HWB 002-11
Flasks with vented caps	Dutscher	cat.no. 355121B
Gavage syringe	Fine Science Tools	cat.no. 18061-22
MicroAmp EnduraPlate Optical 96-Well plates	ThermoFisher Scientific	cat.no. 4483485
Parafilm	Merck	cat.no. 291-1213
PCR tube	Eppendorf	cat.no. 30124359
Petri Dish	Dutscher	cat.no. 633180
Pre-Separation Filter	Miltenyi Biotec	cat.no. 130-041-407
Reservoir for multichannel pipettes	Sigma	cat.no. 4870
Stericup	Millipore	cat.no. S2GPU05RE
Tube DNA LoBind 0.5 ml	Merck	cat.no. EP0030108035
Tube DNA LoBind 1.5 ml	Merck	cat.no. EP0030108051

### Software

**Table:** 

Reagent or resource	Source	Identifier
Adobe Illustrator CC 2019	Adobe	https://www.adobe.com/fr/products/illustrator.html
Blastn	NCBI	https://blast.ncbi.nlm.nih.gov/
BD FACS sort	BD Biosciences	Not Available
FlowJo v10.8	BD Biosciences	https://www.flowjo.com/
ggplot2	Not Applicable	https://ggplot2.tidyverse.org/
GraphPad Prism 9	GraphPad Software	https://graphpad.com/scientific-software/prism/
IgBlast	NCBI	https://www.ncbi.nlm.nih.gov/igblast/
SnapGene	SnapGene	https://www.snapgene.com
MS Excel 2016	Microsoft	https://www.microsoft.com/fr
Adobe Photoshop CC 2019	Adobe	https://www.adobe.com/fr/products/photoshop/landpb.html
FB5P-seq	Not Applicable	https://github.com/MilpiedLab/FB5P-seq
Seurat v4	Not Applicable	https://satijalab.org/seurat/

## Methods

### Mouse model

*Aicda-Cre-ERT2 x Rosa26-lox-STOP-lox-eYFP* mice (20) were bred at the Centre d’Immuno-Phenomique, (Marseille, France), and transferred to the animal care facility of Centre d’Immunologie de Marseille-Luminy for experiments. All mice were maintained in the CIML mouse facility under specific pathogen-free conditions. Experimental procedures were conducted in agreement with French and European guidelines for animal care under the authorization number APAFIS #30945-2021040807508680, following review and approval by the local animal ethics committee in Marseille. Mice were used regardless of sex, at ages greater than 7 weeks and less than 3 months.

**Figure 2:**
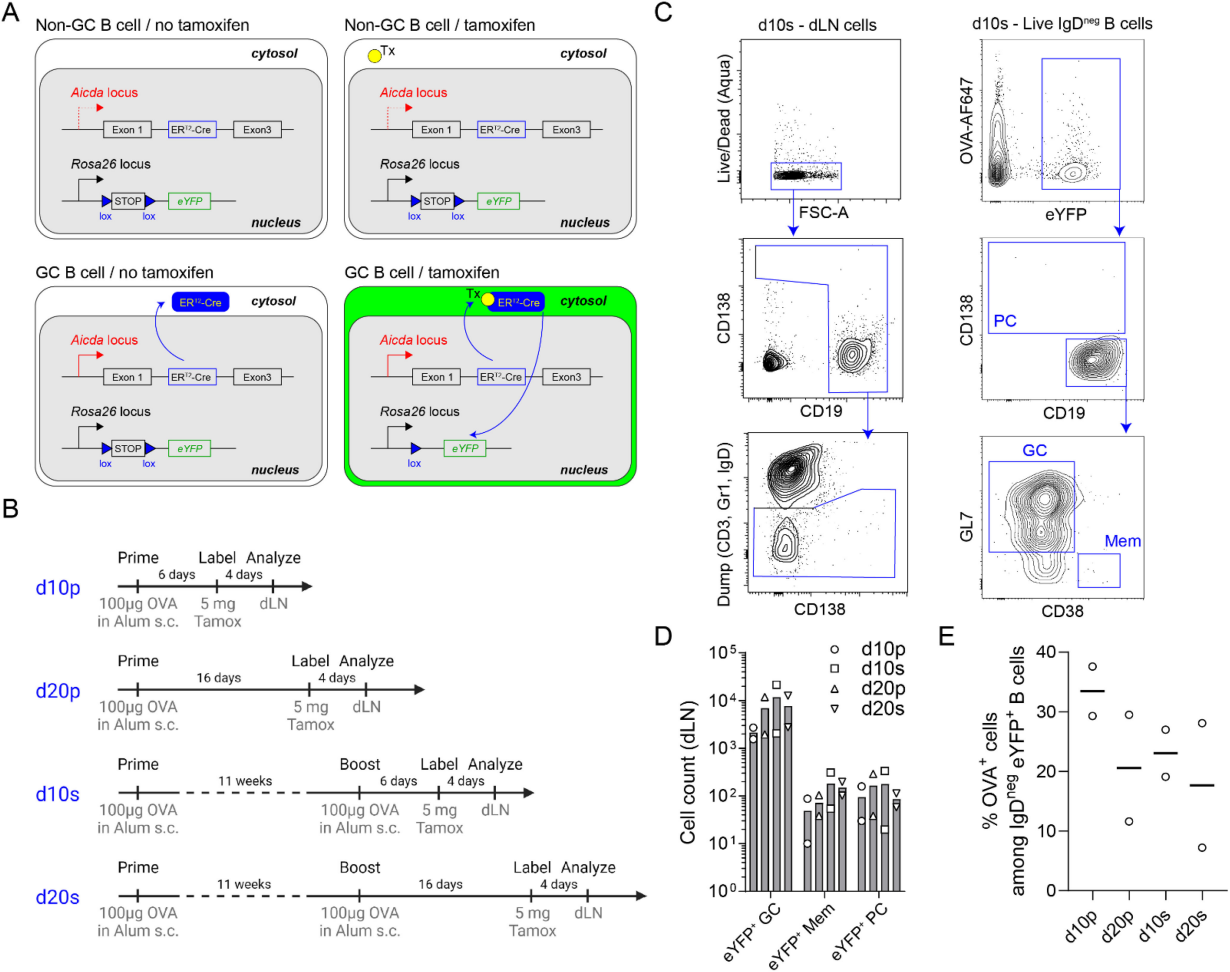
Experimental design for OVA-specific GC B cell analysis by FB5P-seq-mAbs. **(A)** Schematic description of the Aicda-Cre-ERT2^+/-^ x Rosa26-eYFP-lox-stop-lox^+/-^ mouse model for fate mapping GC B cells and their progeny after tamoxifen gavage of immunized mice. **(B)** Immunization strategies used to study GC B cells and their progeny at day 10 or day 20 after primary or secondary immunizations. **(C)** Full gating strategy for fate mapped GC B cells and their memory B cell (Mem) and plasma cell (PC) progeny in draining lymph nodes. **(D)** Absolute counts of eYFP^+^ GC B cells, eYFP^+^ Mem and eYFP^+^ PC in draining lymph nodes in d10p, d10s, d20p and d20s animals. **(E)** Percentage of OVA^+^ cells among IgD^neg^ eYFP^+^ B cells in draining lymph nodes in d10p, d20p, d10s and d20s animals.

Mice were immunized with 100µg chicken ovalbumin (OVA) at 1µg/µl emulsified with Alum at a 1:1 (v:v) ratio, subcutaneously at the base of the tail, 50µl on each side. For induction of the Cre-ERT2-mediated labelling, we gavaged the mice once with 5mg of tamoxifen (TS648-1G, Sigma) in 200µL of peanut oil (P2144-250 ML, Sigma), at least 6 days after immunization. Mice were euthanized between 10 days and 21 days post-immunization (prime or boost) according to the experiment (**Figure 2B**).

### Flow cytometry and cell sorting of B cell subsets

Single-cell suspensions from draining lymph nodes were washed and resuspended in FACS buffer (5% fetal calf serum, 2mM EDTA, 5% Brilliant Stain Buffer Plus in PBS 1X) at a concentration of 100 million of cells per ml. For each dLN cell suspension, we stained 5 million cells in a final volume of 100 µl (50 µl of cells at a 100 x 10^6^ cells/ml concentration, plus 50 µl of antibody mix). We used single-color compensation controls to verify the settings of the cytometer on the day of the sort, running the experiment on a template that had been previously established according to good cytometry practice. Cells were first incubated with FcBlock (Biolegend) for 10 min on ice. Then, cells were incubated with a mix of antibodies conjugated with fluorochromes 30 min on ice. Cells were washed in PBS, and incubated with the Live/Dead Fixable Aqua Dead Cell Stain (Thermofisher) for 10 min on ice. Cells were then washed again in FACS buffer and resuspended in FACS buffer. Cells were sorted on the BD Influx™ Cell Sorter, in 96-well plates, with index-sorting mode for recording the fluorescence parameters associated to each sorted cell.

### FB5P-seq library preparation, sequencing and data pre-processing

The protocol was performed as previously described by Attaf et al. (9). Individual cells were sorted into a 96-well PCR plate, with each well containing 2 µL of lysis buffer. Index sort mode was activated to record the fluorescence intensities of all markers for each individual cell. Flow cytometry standard (FCS) files from the index sort were analyzed using FlowJo software, and compensated parameters were exported as CSV tables for subsequent bioinformatic analysis. Immediately after sorting, plates containing individual cells were stored at -80°C until further processing. Following thawing, reverse transcription was performed, and the resulting cDNA was preamplified for 22 cycles. Libraries were then prepared according to the FB5P-seq protocol. The FB5P-seq data were processed to generate both a single-cell gene count matrix and single-cell B cell receptor (BCR) repertoire sequences for B cell analysis. Two separate bioinformatic pipelines were employed for gene expression and repertoire analysis, as detailed in Attaf et al. (9).

### Bioinformatics analysis

We used a custom bioinformatics pipeline to process fastq files and generate single-cell gene expression matrices and BCR sequence files as previously described (9). Detailed instructions for running the FB5P-seq bioinformatics pipeline can be found at https://github.com/MilpiedLab/FB5P-seq. Quality control was performed on each dataset independently to remove poor quality cells based on UMI counts, number of genes detected, ERCC spike-in quantification accuracy, and percentage of transcripts from mitochondrial genes. For each cell, gene expression UMI count values were log-normalized with Seurat *NormalizeData* with a scale factor of 10,000 to generate normalized UMI count matrices.

Index-sorting FCS files were visualized in FlowJo software and compensated parameters values were exported in CSV tables for further processing. For visualization on linear scales in the R programming software, we applied the hyperbolic arcsine transformation on fluorescence parameters (21).

For BCR sequence reconstruction, the outputs of the FB5P-seq pipeline were further processed and filtered with custom R scripts. For each cell, reconstructed contigs corresponding to the same V(D)J rearrangement were merged, keeping the largest sequence for further analysis. We discarded contigs with no constant region identified in-frame with the V(D)J rearrangement. In cases where several contigs corresponding to the same BCR chain had passed the above filters, we retained the contig with the highest expression level. BCR metadata from the *MigMap* and *Blastn* annotations were appended to the gene expression and index sorting metadata for each cell.

Supervised annotation of scRNA-seq datasets were performed as described extensively in Figure 2B and Supplementary Figure 2C of Binet et al. (22). Briefly, we used the *AddModuleScore* function to compute gene expression scores for every cell in the dataset for the cell type specific signatures. For DZ and LZ signatures, genes associated to cell cycle ontologies (based on GO terms), were removed from the gene lists prior to scoring, as described in Milpied et al. (23). Thresholds for “gating” were defined empirically. Single-cell gene expression heatmaps were generated by the *doheatmap* function in *R*.

### BCR amplification and cloning

From the FACS-sorted single-cell RNA-seq libraries in 96-well PCR plates, 2 µl of each well of the plate was diluted. This diluted cDNA was used to amplify the variable regions with a multiplex PCR (**Supplementary Table 1**). After verification of the amplification on an agarose gel, the PCR products were purified and adjusted to the concentration necessary to perform the cloning (40 ng/µl). Cloning of the variable regions was done by SLIC (Sequence and ligation-independent cloning) in 1 µl of each linearized and pre-purified expression vector concentrated at 40 ng/µl (24). To verify that the inserts had been cloned into each expression vector, the colonies were screened by PCR and a bacterial colony fingerprint was made. The primers used were specific to each IgH and IgK vector (**Supplementary Table 2**). The two vectors used in this study were kindly provided by the Nussenzweig laboratory (15).

Plasmid DNA preparations were made from the screened positive bacterial fingerprints and each construct was sequenced and checked against the original sequences.

### Antibody production

The Expi293™ cells grown in Expi293™ Expression Medium were cultured in sterile vented-cap Erlenmeyer flasks. On the day of transfection, the heavy chain-containing vectors and light chain-containing vectors were incubated in the presence of polyethyleneimine. The cells were incubated for 6 days in the incubator. The culture supernatants were then centrifuged, filtered and the antibodies present in supernatant were purified on protein G beads on polyprep chromatography columns.

### Antibody analysis by ELISA

OVA antigen at 20 µg/ml was coated in 96-well ELISA plates (50 µl per well) for incubation overnight at 4°C. After 3 washes with PBS + Tween 0.05%, the plate was blocked with 100 μl of blocking buffer (PBS + 2% BSA) and incubated for 2h at room temperature. After 3 washes, a stepwise serial dilution (dilution factor 4), starting from 1 µg antibody per well was prepared for control and target antibodies, and was added to the plate wells in two replicates (50 µl per well) and incubated for 2h at room temperature. In all assays, we included a commercial anti-OVA monoclonal antibody (clone 6C8) as a positive control, and no primary antibody (only secondary HRP-conjugated anti-IgG antibody) as a negative control. After 3 washes, HRP-coupled anti-mouse IgG secondary antibody diluted 4000-fold was added and incubated for 2h at room temperature. After 3 washes, 100 µl of TMB solution was added and incubated for 15 min. The reaction was stopped by adding 100 µl of 2N sulfuric acid. The reaction was measured with a plate reader at 450 nm.

### Detailed protocol

A detailed reagent setup protocol is presented in **Supplementary Table 3**.

A detailed step-by-step protocol is presented in **Supplementary Table 4**.

## Limitations

The amplification of the Ig genes may be dependent on the sequence of the V genes sequences of the isolated B cells. It may be necessary to amplify the BCR from a larger number of cells to obtain the sufficient number of Ig. However, the knowledge of each BCR sequence upstream of the amplification, can be a precious help in the failure of some V gene PCR products, by being able to specifically adapt the sequence of the primers.

The cloning of variable chains by the SLIC method can be variable. This can be due to the purification of the PCR products or the efficiency of the reaction. In this case, it is possible to overcome this problem by a classical cloning technique which is made possible by the presence of restriction enzyme sites in the PCR products of each chain.

The Elisa control experiment which is performed the day before antibody purification is a good indication of antibody production in the culture supernatant. However, the level of production does not always reflect the total amount of antibodies obtained after purification. This depends on the ability of the antibodies to bind to the protein-G beads due to their structure or purity.

## Troubleshooting

A detailed troubleshooting table is presented as **Supplementary Table 5**.

## Results

We applied FB5P-seq-mAbs to study murine GC B cells after prime or prime-boost immunization with the model antigen chicken ovalbumin (OVA). We used the Aicda-Cre-ERT2^+/-^ x Rosa26-eYFP-lox-stop-lox^+/-^ mouse model (20, 25) for fate mapping GC B cells and their progeny after tamoxifen gavage of immunized mice (**Figure 2A**). Mice were immunized with OVA and alum adjuvant subcutaneously, once for prime immunizations, a second time 11 weeks later for prime-boost immunizations, and gavaged with tamoxifen 4 days before sacrifice and collection of draining lymph nodes; draining lymph nodes were collected 10 or 20 days after primary (d10p, d20p), or after secondary (d10s, d20s) immunization (**Figure 2B**). GC B cells and their progeny were gated as live IgD-negative B cells expressing eYFP, and were further subdivided as CD138^+^ plasma cells (PC), CD19^+^GL7^+^CD38^-^ GC B cells, or CD19^+^CD38^+^GL7^-^ memory B cells (Mem) (**Figure 2C**). As expected, eYFP^+^ GC B cells outnumbered their Mem and PC progeny at days 10 or 20 after primary or secondary immunization in draining lymph nodes (**Figure 2D**). Among IgD^neg^ eYFP^+^ B cells, OVA-binding cells were a minority, representing an average of 20-30% (**Figure 2E**).

**Figure 3:**
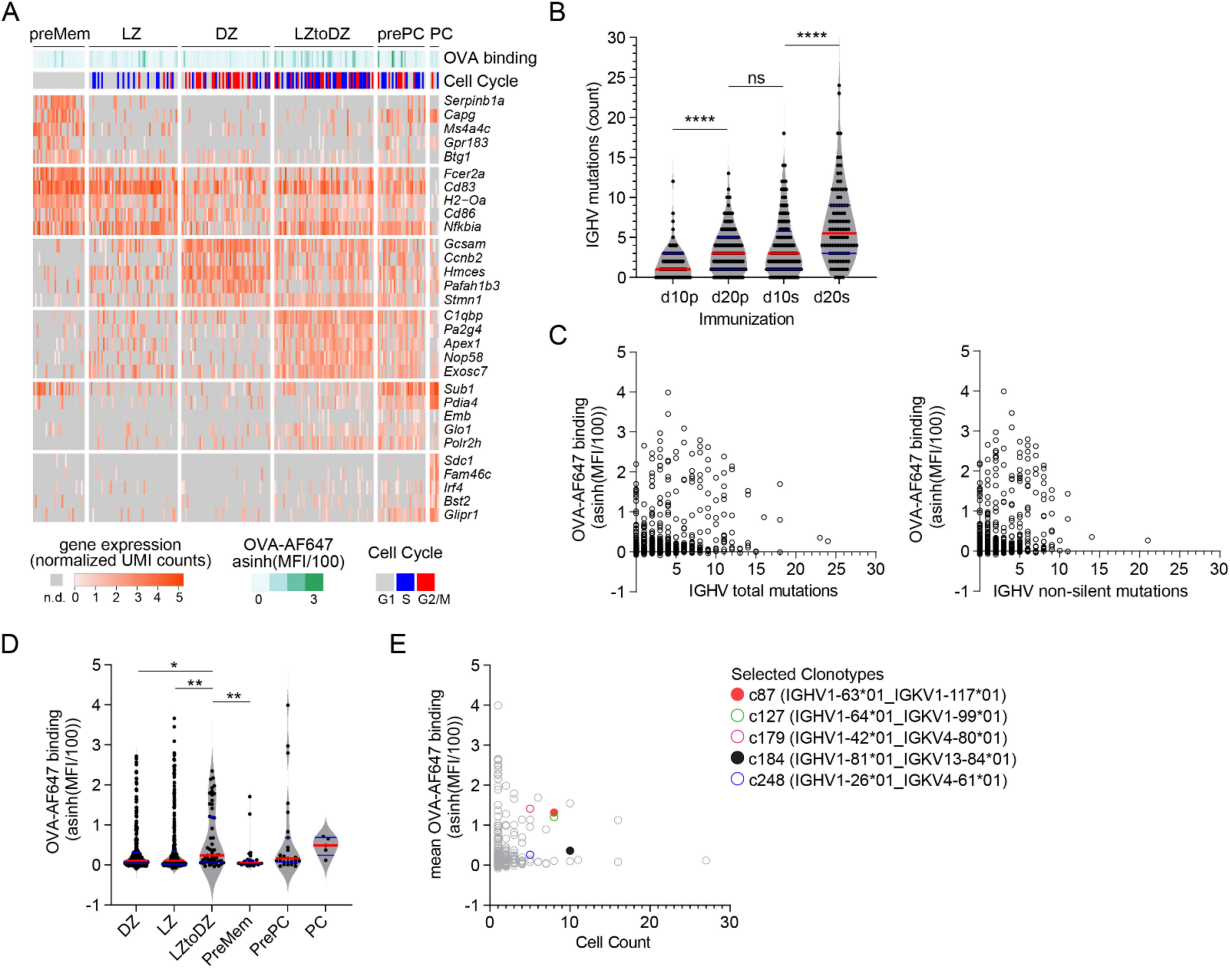
FB5P-seq analysis of OVA-specific GC B cells. **(A)** Gene expression heatmap showing expression of top 5 marker genes for annotated subsets of GC B cells. For visualization, only randomly sampled 50 cells from LZ and DZ subsets are displayed on the heatmap. Bars above the heatmap indicate OVA-AF647 surface binding (fluorescence intensity) and cell cycle phase inferred from gene expression. **(B)** Number of mutations in *Ighv* sequences of cells from d10p, d20p, d10s, and d20s samples, as compared to their inferred germline. Each dot is a cell. ****p<0.0001, ns, not significant, adjusted p-value for Dunn’s multiple comparison test after significant Kruskal-Wallis analysis. **(C)** Scatter plot of *Ighv* total (left) and non-silent (right) mutation count (x-axis) by OVA-AF647 surface binding intensity (y-axis). Each dot is a cell. **(D)** OVA-AF647 surface binding intensity in cells from different gene expression-based subsets. Each dot is a cell. *p<0.05, **p<0.01, adjusted p-value for Dunn’s multiple comparison test after significant Kruskal-Wallis analysis. **(E)** Scatter plot of clonotype size (x-axis) by mean OVA-AF647 surface binding intensity (y-axis) for all clonotypes. Each dot is a clonotype, colored symbols for clonotypes selected for further analyses.

We sorted single IgD^neg^ eYFP^+^ B cells from d10p, d20p, d10s and d20s draining lymph nodes, in order to gain access to GC B cells with distinct levels of affinity maturation and somatic hypermutation, and prepared 5’-end scRNA-seq libraries with the FB5P-seq protocol (9). All surface staining parameters, including binding of OVA-AlexaFluor647 (OVA-AF647) antigen, were recorded by index sorting and appended to the gene-by-cell UMI count matrix. We also used the FB5P-seq bioinformatic pipeline to reconstruct the *Igh* and *Igk/l* variable region sequences from 5’-end scRNA-seq reads (9). After quality controls, we retained 769 cells(d10p: 145 cells; d20p: 205 cells; d10s: 295 cells; d20s: 124 cells), including 573 cells with paired full *Igh* and *Igk/l* variable region sequences (d10p: 101 cells; d20p: 158 cells; d10s: 218 cells; d20s: 91 cells). Based on signature gene expression, we annotated the majority of cells as LZ (n=331), DZ (n=300) or recycling LZtoDZ (n=55) GC B cells, and identified minor fractions of putative preMem (n=29), prePC (n=27), or *bona fide* PC (n=5) (**Figure 3A**). Consistent with other scRNA-seq studies of murine GC B cells (26–28), LZ cells expressed high levels of *Fcer2a* (encoding CD23), *Cd83*, *H2-Oa* (encoding MHC-II subunit), *Cd86* and *Nfkbia* transcripts and were mostly quiescent or in S phase. DZ cells expressed high levels of *Gcsam*, *Ccnb2*, *Hmces*, *Pafah1b3* and *Stmn1* transcripts, and were mostly in G2/M phase. LZtoDZ cells expressed both LZ and DZ marker genes, as well as high levels of *C1qbp*, *Pa2g4*, *Apex1*, *Nop58* and *Exosc7* transcripts, and were mostly in S phase. PreMem cells expressed LZ marker genes, as well as high levels of *Serpinb1a*, *Capg*, *Ms4a4c*, *Gpr183* and *Btg1* transcripts, and were quiescent. PrePC cells were transcriptionally close to LZtoDZ cells, with additional expression of *Sub1*, *Pdia4*, *Emb*, *Glo1*, *Polr2h* transcripts, and were mostly proliferating. PC expressed high levels of some PrePC marker genes (*Sub1*, *Pdia4*) and other transcripts associated with antibody-producing cells differentiation such as *Sdc1* (encoding CD138), *Fam46c*, *Irf4*, *Bst2*, and *Glipr1*.

Consistent with ongoing affinity maturation occurring in primary and secondary GC, the number of *Ighv* mutations increased from d10p to d20p, and then further increased from d10s to d20s (**Figure 3B**). Surface binding of OVA-AF647 antigen, a flow cytometry-based measurement of BCR affinity for the immunizing OVA antigen, was not correlated to the number of *Ighv* total or non-silent mutations (**Figure 3C**). Among the different cell subsets, recycling LZtoDZ cells had the highest rate of OVA-AF647 binding (**Figure 3D**), consistent with high affinity antigen-specific cells being selected and recycled from LZ GC B cells at all stages of primary and secondary GC reactions. We used *Ighv* sequence information to identify groups of clonally related cells (clonotypes). We retrieved 291 distinct clonotypes, including 188 unique clones and 103 clonotypes with two or more cells. We randomly selected 5 clonotypes among clonotypes of size ≥5 containing at least 1 cell with OVA-AF647 surface binding above background (**Figure 3E**) for recombinant mAb expression. Those clonotypes were from d10p (c127, c184, c248) and d10s (c87, c179) animals.

For each of those selected clonotypes, we designed unmutated germline sequences for the *Ighv* and *Igkv* chains, and had them synthesized with appropriate 5’ and 3’ ends for direct cloning into our IgG1 and IgK expression vectors. All other *Ighv* and *Igkv* sequences were amplified and cloned directly from the archived single-cell cDNA obtained in the FB5P-seq protocol, as detailed in the methods. We succeeded to obtain significant amounts of recombinant mAb for 5/5 cells for clonotype c179, 5/5 cells for clonotype c248, 7/8 cells for clonotype c127, 7/8 cells for clonotype c87, and 8/10 cells for clonotype c184. Failures were due to unsuccessful PCR for one of the two chains of a given cell. In some cases where the multiplexed PCR failed, we used the reconstructed *Ighv* sequence from the FB5P-seq pipeline to design sequence-specific forward primers for *Ighv* amplification, and obtained PCR products that were cloned and used to produce mAbs.

**Figure 4:**
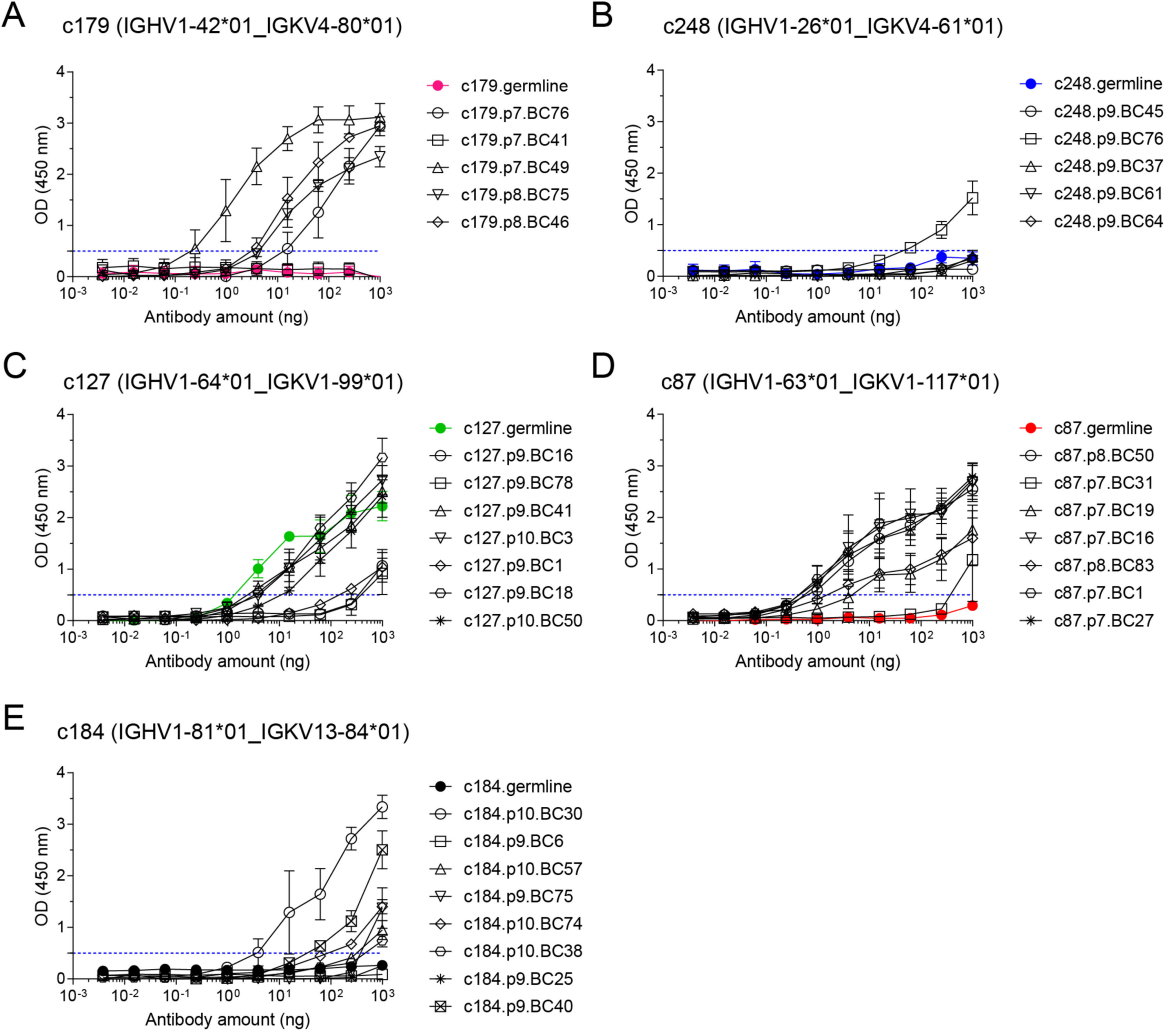
OVA binding capacity of recombinant mAbs measured by indirect ELISA assay. Recombinant mAbs from clonotypes c179 **(A)**, c248 **(B)**, c127 **(C)**, c87 **(D)**, and c184 **(E)** and their respective germline (colored line and symbols in each plot) were tested as serial 4-fold dilutions (starting from 1µg per well) in indirect ELISA on OVA-coated plates. All measurements were performed independently from 2 to 5 times, and mean ± s.e.m. OD values are reported as connected symbols. The dotted blue line represents the threshold OD value that was used to compute the ELISA Binding Threshold value for each mAb.

**Figure 5:**
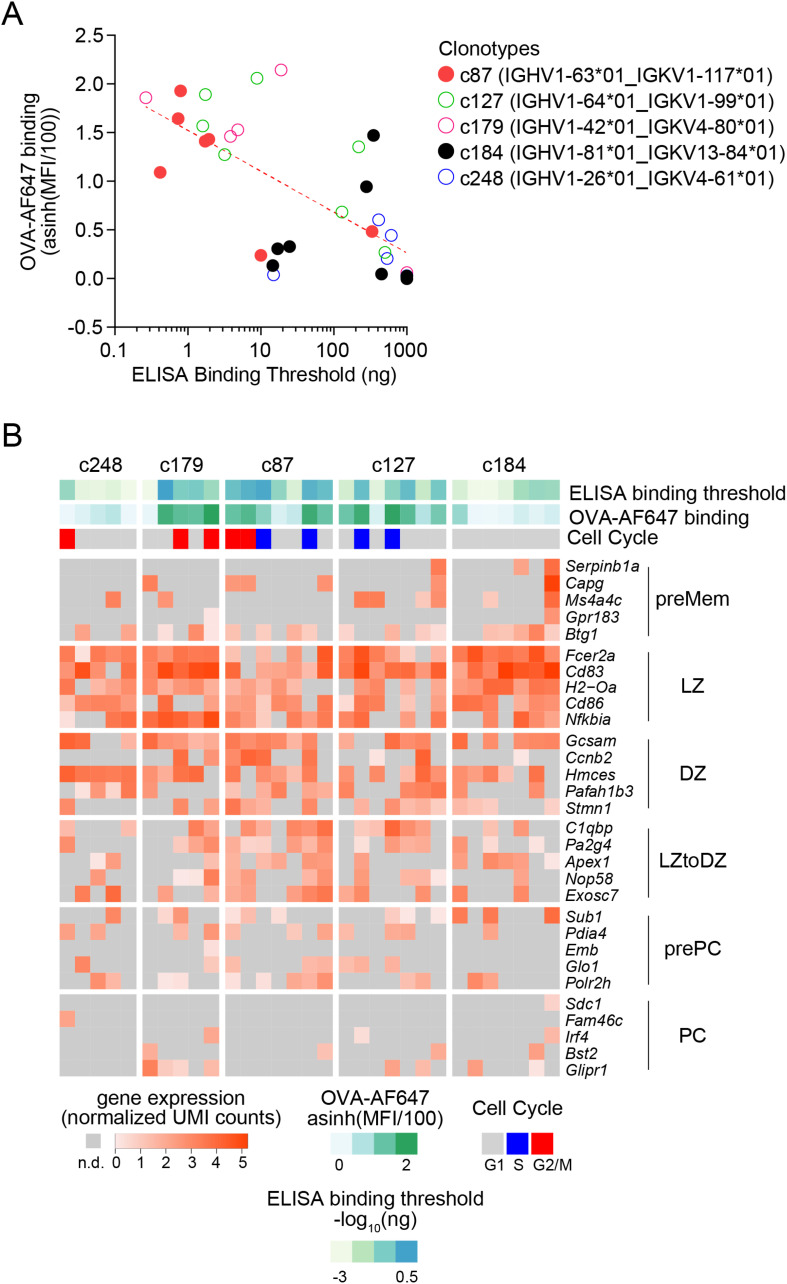
Integrative analysis of antigen-binding and gene expression of OVA-specific GC B cells. **(A)** Scatter plot of ELISA Binding Threshold (x-axis) by OVA-AF647 binding (y-axis) for mAbs of the indicated clonotypes (color key). The dashed red line indicates the non-linear semi-log least-squares fit (R^2^= 0.4638). **(B)** Gene expression heatmap showing expression of top 5 marker genes of subsets of GC B cells (as in **Figure 3A**) in cells from the selected clonotypes studied by ELISA. Bars above the heatmap indicate OVA ELISA Binding Threshold, OVA-AF647 surface binding (fluorescence intensity) and cell cycle phase inferred from gene expression.

We then tested serial dilutions of those mAbs for OVA binding in indirect ELISA assays (**Figures 4A–E**). For all clonotypes, at least one mAb detectably bound OVA, albeit at very high concentration for clonotype c248 (**Figure 4B**). As expected, there was intraclonal heterogeneity in antigen binding capacity of single-cell mAbs, with most mAbs binding better than the germline for all clonotypes except c127 (**Figures 4A–E**), but some mAbs having no detectable OVA binding for clonotypes c179 (**Figure 4A**), c248 (**Figure 4B**), c87 (**Figure 4D**) and c184 (**Figure 4E**). For clonotype c127, none of the GC B cell-derived mAbs bound better than the germline (**Figure 4C**). Based on the ELISA binding curves, we computed the ELISA Binding Threshold values for all mAbs, reflecting the minimum amount of mAbs giving detectable binding in our ELISA assays. There was no association between the number of mutations in the *Ighv* or *Igkv* regions and the ELISA Binding Threshold (**Table 1**), but there was a significant correlation between the ELISA Binding Threshold and the OVA-AF647 surface binding measured by index sorting (**Table 1**, **Figure 5A**), even for intraclonal comparisons. Most cells which had surface binding of OVA-AF647 above 1, corresponding to signal above background noise, produced mAbs with ELISA Binding Threshold below 10 ng. Conversely, most cells which had surface binding of OVA-AF647 below 1 produced mAbs with ELISA Binding Threshold above 100 ng. Thus, OVA-AF647 surface binding is a good proxy for assessing antigen-binding properties of the BCR in single GC B cells. Nevertheless, 5 outlier cells, 3 of which were from clonotype c184, had no detectable surface binding of OVA-AF647, but produced mAbs with ELISA Binding Threshold values between 10 and 100 ng, suggesting that the FACS assay may be less sensitive than the ELISA on recombinant mAbs. We inspected the expression of subset-specific marker genes in individual cells in relation with the binding capacities of their BCR measured by FACS and ELISA (**Figure 5B**). Consistent with our observations on the total dataset (**Figure 3D**), it was interesting to note that for clonotypes c179, c87 and c127, most of the cells with good OVA-binding capacity expressed genes associated with LZ, DZ, and LZtoDZ states. For both clonotypes c127 and c184, we captured one cell in PreMem state that expressed OVA-binding BCR with ELISA Binding Threshold values in the 10 ng range, consistent with the selection of Mem B cells from mid-affinity GC B cells (29).

**Table 1:** Characteristics of recombinant mAbs produced from OVA-specific GC B cell clonotypes.

	number of mutations				ELISA binding threshold (ng)	OVA-AF647 binding (asinh(MFI/100))
	VH		VK			
	CDR	FW	CDR	FW		
c179 (IGHV1-42*01_IGKV4-80*01)						
germline	0	0	0	0	>1000	Not Available
p7.BC76	1	0	1	0	19.03	2.14
p7.BC41	0	1	2	1	>1000	0.06
p7.BC49	0	1	0	0	0.27	1.86
p8.BC75	1	0	1	0	4.85	1.53
p8.BC46	0	0	0	0	3.86	1.46
c248 (IGHV1-26*01_IGKV4-61*01)						
germline	0	0	0	0	>1000	Not Available
p9.BC45	2	3	0	0	>1000	0.03
p9.BC76	2	1	0	0	14.94	0.04
p9.BC37	2	1	0	0	615.18	0.44
p9.BC61	0	1	0	0	539	0.21
p9.BC64	0	0	0	0	412	0.60
c127 (IGHV1-64*01_IGKV1-99*01)						
germline	0	0	0	0	0.75	Not Available
p9.BC16	1	1	0	2	219	1.35
p9.BC78	1	0	0	0	502	0.27
p9.BC41	1	1	0	1	1.74	1.89
p10.BC3	0	0	0	0	1.6	1.57
p9.BC1	1	0	0	0	129	0.68
p9.BC18	0	0	0	0	3.2	1.27
p10.BC50	1	1	0	0	8.9	2.06
c87 (IGHV1-63*01_IGKV1-117*01)						
germline	0	0	0	0	>1000	Not Available
p8.BC50	2	5	2	3	0.8	1.93
p7.BC31	2	1	0	1	336	0.48
p7.BC19	1	2	0	2	10.8	0.24
p7.BC16	1	3	1	0	1.94	1.43
p8.BC83	2	1	1	1	1.73	1.41
p7.BC1	0	0	1	0	0.74	1.64
p7.BC27	0	0	0	0	0.42	1.09
c184 (IGHV1-81*01_IGKV13-84*01)						
germline	0	0	0	0	>1000	Not Available
p10.BC30	1	1	0	4	14.6	0.13
p9.BC6	2	0	2	2	>1000	0.03
p10.BC57	0	0	0	0	352	1.47
p9.BC75	1	1	0	0	451	0.05
p10.BC74	1	0	0	4	24.8	0.33
p10.BC38	1	1	0	0	280	0.94
p9.BC25	1	2	0	0	>1000	0.00
p9.BC40	2	0	0	0	17	0.31

For all mAbs grouped by clonotype, the number of mutations in the CDR or framework (FW) regions of the heavy (VH) and light (VK) chains are indicated in the first 4 columns, the ELISA Binding Threshold in ng in the 5^th^ column, and the OVA-AF647 surface binding measured by index sorting in the 6^th^ column.

Altogether, those results illustrate some of the quantitative single-cell analyses that are made possible when integrating phenotypic, transcriptomic, molecular and biochemical readouts on single B cells with FB5P-seq-mAbs.

## Discussion

FB5P-seq-mAbs was designed as an extension of our plate-based 5’-end scRNA-seq method, FB5P-seq (9). As such, it can be performed on archived single-cell cDNA months to years after preparation of the initial FB5P-seq libraries. Here, we demonstrated FB5P-seq-mAbs on mouse OVA-specific GC B cells, but it could also be applied to other antigen-specific B cell types. For example, we recently applied FB5P-seq to characterize memory B cells in mouse lungs after influenza virus infection and discovered bystander Mem B cells with no apparent specificity to influenza virus antigens (11). In that case, FB5P-seq-mAbs may be useful to produce recombinant mAbs from bystander Mem B cells and screen those mAbs for other (auto)antigen specificities.

The FB5P-seq-mAbs protocol can be easily adapted to study human B cells by using the *IGH* and *IGK/L* amplification and cloning strategies described by others previously (13). In FB5P-seq-mAbs, the scRNA-seq and BCR sequence reconstruction are performed before cloning of *IGH* and *IGK/L*, giving the opportunity to select only the single-cell cDNAs from specific cell states and/or clonotypes as starting material for mAb production. Knowing the *IGH* and *IGK/L* sequences before cloning is also particularly interesting when working with highly mutated B cells, because it allows the design of cell-specific or clonotype-specific PCR primers instead of multiplexed PCR primers that may not be optimal.

FB5P-seq-mAbs is modular, and equivalent integrative single-cell analyses of B cells may be obtained via distinct alternatives. Other plate-based single-cell RNA-seq library preparation protocols, such as Smart-seq2 (7) or Smart-seq3 (30), may be used to produce single-cell gene expression and BCR sequencing data and archive single-cell cDNA for recombinant mAb production. Other cloning and expression methods may be used to produce recombinant mAbs from single-cell cDNA (31). Another possibility is to use high-throughput droplet-based methods for characterization of phenotype, gene expression, BCR sequence and antigen binding (12), then select cells of interest *in silico* and have their *IGH* and *IGK/L* V genes synthesized for direct cloning and production.

The two main directions for improving FB5P-seq-mAbs are depth and throughput. First, we may modify the FB5P-seq protocol to adopt recent improvements to plate-based scRNA-seq protocols that make them more sensitive and easier to perform. For example, we may implement the one-step RT-PCR of the recently published FLASH-seq-UMI protocol (32) which resulted in higher sensitivity and shorter library preparation time when compared to the Smart-seq3 approach. Second, we may implement antibody cloning and production strategies that are designed for higher throughput (33, 34) and can be automatized.

FB5P-seq-mAbs and its future improved versions will be important to continue the in-depth studies of antigen-specific B cell responses in animal models, and to discover the antigen reactivity and affinity of B cells in human infectious diseases, autoimmunity and cancer.

## Data Availability

Publicly available datasets were analyzed in this study. This data can be found here: NCBI GEO accession number GSE275121.
