# Supplementary material for: FB5P-seq-mAbs: monoclonal antibody production from FB5P-seq libraries for integrative single-cell analysis of B cells
Source: Front Immunol. 2024 Dec 17;15:1505971. doi: 10.3389/fimmu.2024.1505971 (PMC11685048; doi:10.3389/fimmu.2024.1505971)
Supplement: Supplementary file 2 [file Table2.docx]

**Supplementary Table 2 : Primers for screening PCR and sequencing**

| Screening Forward Primers (IgG1 and IgK) | GCTTCGTTAGAACGCGGCTAC |
| --- | --- |
| Screening Reverse Primers (IgG1) | GGGTCACCATGGAGTTAGTTTGG |
| Screening Reverse Primers (IgK) | TCCACTTGACATTGATGTCTTTGG |
